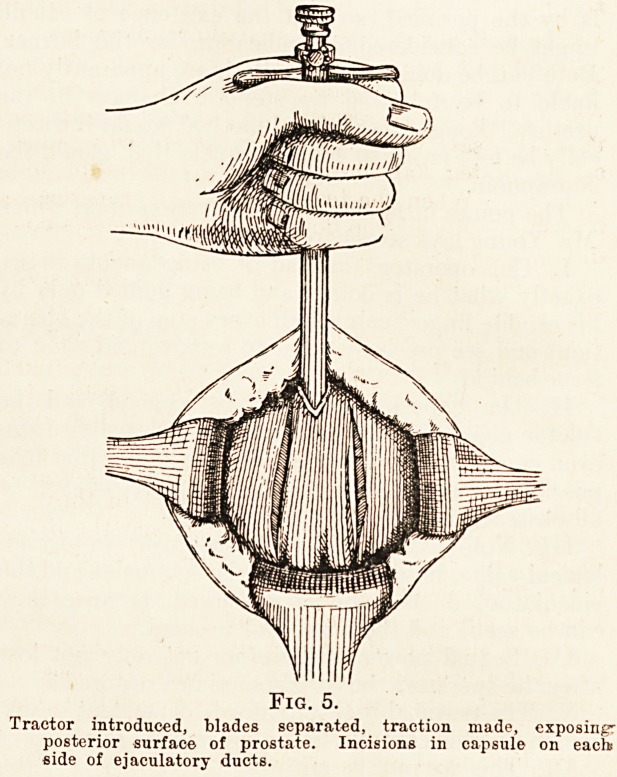# Perineal Prostatectomy

**Published:** 1908-04-11

**Authors:** 


### SPECIAL ARTICLE.

### PERINEAL PROSTATECTOMY.

It is quite obvious that many persons who have enlargement of the prostate require no treatment for the condition at all. It is not every big prostate that obstructs the outflow of the urine from the bladder; but when such obstruction does result, as it does unfortunately in a great many cases, it is clear that one or other of three main courses are open:—

I. To do nothing, at any rate for the time being, the surplus urine being evacuated by the patient, and a residuum constantly remaining.

II. To evacuate the residual urine from time to time by means of a catheter.

III. To remove the obstructing prostate by means of an operation.

There is no need to say much about the risks of Courses I. and II.; frequency of micturition, especially at night, with disturbed rest and consequent deterioration of the general health, is one of the most certain results of Course I.; whilst decomposition of the urine, cystitis, and ascending nephritis are almost certain to follow both I. and II. sooner or later. It is impossible to follow Course III. in some cases, for monetary reasons, or because of the remoteness of the locality in which the patient lives, and so forth; but it is generally allowed nowadays that the ideal treatment for the trouble is prostatectomy, and prostatectomy quite early before the strength of the elderly patient has been sapped by broken rest at night, and by the misery of cystitis.

It is for the patient to decide, of course, whether he will be operated upon or not; and it very frequently happens that he postpones the time for operation until his sufferings have become considerable; by then the risks of the operation have been materially increased. Nevertheless, even though the operation of prostatectomy has been done, perforce, upon subjects whose general health has deteriorated far below what it need have done, the statistics upon the mortality of the procedure show that the operative risks are comparatively slight notwithstanding the advanced age of the patients.

General practitioners who have once seen the immense improvement in health, the rejuvenescence of some of their oldest patients after prostatectomy, are loud in their praises of the operation; their patients are saved to them for many years longer than they otherwise would have been. The advisability of an operation for prostatectomy is, therefore, not in question; the purpose of this article is, however, to lay stress upon the fact that prostatectomy can be done in more than one way.

In England the fashion at present is to perform enucleation of the prostate by suprapubic cystotomy; and there is no doubt that the method is a most excellent one. It has disadvantages, however, and one of the gravest of these is the difficulty in getting the patients off their backs soon after it. It is a very serious matter for an elderly gentleman, after an anæsthetic, to be compelled to be more or less upon his back for a time. The risk of hypostatic pneumonia is considerable.

Mr. Hugh H. Young, of Johns Hopkins University, has performed prostatectomy by different methods in a great number of cases, and he has quite come to the conclusion that, when all the advantages and disadvantages are carefully considered and weighed, the best of all methods is that which he calls *conservative perineal prostatectomy*. He gives a full description of the technique of the operation, pointing out each different step, and the procedure is not very difficult.

The operation takes rather longer than does that by suprapubic cystotomy, but the difference is one of minutes only. Mr. Young has made no great attempt to work with extreme rapidity, and yet the time consumed from the first incision to the tying of the last suture after placing the tube and gauge drainage in position varies from fifteen to thirty minutes, twenty-two minutes being about the average. The time during which the anæsthetic is being administered is less than this, the patient being placed on the table as soon as anæsthesia is complete, and the ether being removed considerably before the end of the operation. Spinal anæsthesia was successfully employed in some of the patients.

It will probably be a question, in most cases, of which operation the surgeon is the more familiar with when he decides which he is going to do in any particular case; a surgeon who is highly skilled in the suprapubic method and is unfamiliar with the perineal will probably get a better result by continuing with the former than he would by trying the latter instead; and *vice versâ*. Nevertheless it is important to realise the points which have been urged in favour of the perineal method, seeing that it is so little in fashion in England.

Both methods will relieve the obstruction to the bladder outflow; both will allow of the removal of vesical calculi should any be present at the same time as the large prostate. The removal of calculi is probably more easy by the suprapubic method than it is by the perineal, so that the existence of calculi might be considered an indication for the former. Both can be done quickly. Both are apparently not liable to be followed by stenotic changes in the urethra, though the perineal method would theoretically be less prone to such a sequela than would the suprapubic.

The points in favour of his operation upon which Mr. Young lays stress are as follows:—

I. The operator, instead of being unable to see exactly what he is doing, and being guided only by his erudite finger, can watch every step of the operation, and see precisely what to remove and what to leave behind.

II. The exposure afforded is so good, and the tractor gives such an easy means of drawing down even considerable intra-vesical projections, that it is possible to be quite sure of the complete removal of all obstruction.

III. Non-obstructive structures of certain physiological value, notably the vesiculæ seminales and the ejaculatory ducts, can be preserved, because they can be seen, and their removal avoided.

IV. Sexual power is therefore not only not lost after the operation, but it is sometimes restored by it.

V. The perineal incision affords dependent drainage, in marked contrast to the suprapubic method.

VI. The wound is entirely extra-vesical; the urethra is not damaged.

VII. The wound can be packed easily, and hæmorrhage can be very readily controlled, whereas the control of hæmorrhage is by no means easy in the suprapubic operation.

VIII. All packs and drainage tubes can be removed very early, usually on the day after the operation.

IX. No subsequent manipulations, by means of sounds or catheters, are required.

X. It is easy to remove some malignant prostates completely by the perineal route, whereas it is not so easy to be sure that every part of a malignant prostate has been removed by the suprabubic method. More prostates are malignant than used to be believed; for a long time they do not infiltrate beyond the capsule of the gland, and it is most important to have visual evidence that no part of a malignant prostate is left behind. Mr. Young has several patients who have lived for some years after removal of a cancerous prostate by the perineal route. It is not possible to say beforehand, especially in those cases which are caught as early as they should be, whether the prostate is malignant or merely enlarged, so that the possibility of the existence of malignant disease is a good argument in favour of the perineal method when prostatectomy has been decided.

XI. Last, but very far from least, comes the fact that patients after the operation of perineal prostatectomy can be got out of bed on to a couch in a day or two after the operation, owing to the position of the wound; such early moving of the patient is a most important point, because it has more than anything else has to do with the prevention of pneumonia and of uræmia.

The Johns Hopkins Hospital Reports contain an account of the operation in 185 consecutive cases,

Fig. 1.
The inverted-V cutaneous incision.

some of the patients being nearly 90 years of age, and amongst all this number of unselected cases, only seven died; since then there have been 91 more patients operated upon for prostatectomy by the perineal method without a single death.

### The Technique of the Operation.

There is no very easily obtainable account of the operation to be got, so that we propose to give a description of it as far as possible in Mr. Young's own words.

The exaggerated dorsal lithotomy position is the most satisfactory. The perineal board of the Halstead table is admirably suited for this purpose. The

Fig. 2.
Opening of urethra on sound, preparatory to introduction of tractor.

perineum should be so elevated that it is almost parallel with the floor. Before placing the patient on the table a sound, required for subsequent urethrotomy, should be placed in the urethra as it is difficult to introduce it after the thighs have been flexed.

An inverted V-shaped incision is the best. The apex is taken just over the posterior part of the bulb, and the two branches are each two inches long, the posterior limits being about midway between the anus and the isthial tuberosities (Fig. 1). This incision is carried through the skin, fat, and superficial fascia. The handle of the scalpel is then used on each side of the central tendon to open up the space behind the bulb and in front of the levator ani muscles. This blunt dissection should be carried well down behind the triangular ligament on each side, before any muscular structures are cut. It is easily accomplished, and a good exposure simplifies the next step in the operation.

After exposure of the central tendon by blunt dissection, a bifid retractor is inserted (see Fig. 2), and traction upon it gives an excellent exposure of the narrow band of central muscle and greatly facilitates its division close to the bulb. Great care should be taken not to puncture the bulb—an accident which leads to inconvenient hæmorrhage. After the central tendon has been completely divided a retractor may be placed beneath the bulb, thus affording a better view of the recto-urethralis muscle which lies beneath the two branches of the levator ani and covers the membranous urethra and the apex of the prostate in the median line. Special retractors have been devised for making the different stages of the operation easier than might be the case with ordinary instruments.

At this stage it is generally best to remove the bifid retractor and to insert a narrow-bladed retractor about two inches in depth, by which the rectum can be pushed back and the muscular fibres surrounding the membranous urethra—the recto-urethralis—put under tension. They are then divided by a transverse incision close up to the triangular ligament

Fig. 3.
The prostatic tractor, closed.

Fig. 4.
The prostatic tractor, opened.

and the membranous urethra exposed by blunt dissection.

After the membranous urethra has thus been exposed a retractor is inserted and the apex of the prostate brought into view (Fig. 2). The membranous urethra is then opened on the sound which was inserted into the urethra before the patient was put in the lithotomy position, and the edges of the urethral orifice caught up by silk sutures or by Halsted clamps. A sound of moderate size is then passed through the incision into the prostatic urethra bladder, and the sphincter dilated by a to-and-fro movement of the instrument. The prostatic tractor, closed (Fig. 3), is then passed into the bladder, the edges of the urethral wound being held open by the silk sutures or clamps to facilitate its introduction. Carelessness in this part of the operation may lead to considerable trouble. As soon as the beak is free in the vesical

Fig. 5.
Tractor introduced, blades separated, traction made, exposing posterior surface of prostate. Incisions in capsule on each side of ejaculatory ducts.

cavity the thumb-screw which fixes the blades in position is loosened, the blades rotated 180 degrees by means of the external blades (Fig 4) and then fixed by tightening the thumb-screw again. The instrument is now ready for whatever traction may be necessary to draw the prostate well down into the perineal wound.

Lateral retractors are so placed that, with the posterior retractor drawing the rectum backward and the prostatic tractor drawing the gland outward, a splendid exposure of the entire posterior surface of the prostate is obtained. An incision is then made on each side of the median line for almost the entire length of the posterior surface of the prostate and about three-fifths of an inch deep. The two lines are divergent, as shown in Fig 5, being about three-quarters of an inch apart behind, and three-fifths of an inch apart in front.

The bridge of tissue which lies between them contains the *ejaculatory duct*, and its preservation is of importance if the integrity of these non-obstructive structures is to be left uninjured. It is for this purpose that the initial capsular incisions are three-fifths of an inch deep on each side, as these define at once, and correctly, the width of the "ejaculatory bridge" and prevent its being torn, as might happen if blunt dissection were depended on. These incisions bring one at once to the side of the urethra, where the separation of urethra from inner surface of the prostate can be easily accomplished later on.

(*To be continued.*)

## Figures and Tables

**Fig. 1. f1:**
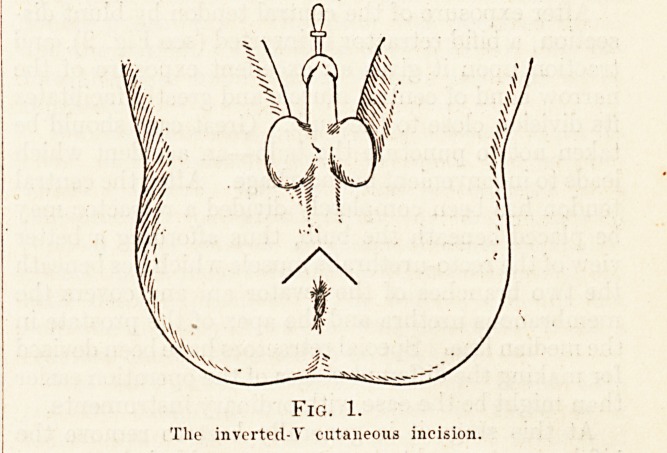


**Fig. 2. f2:**
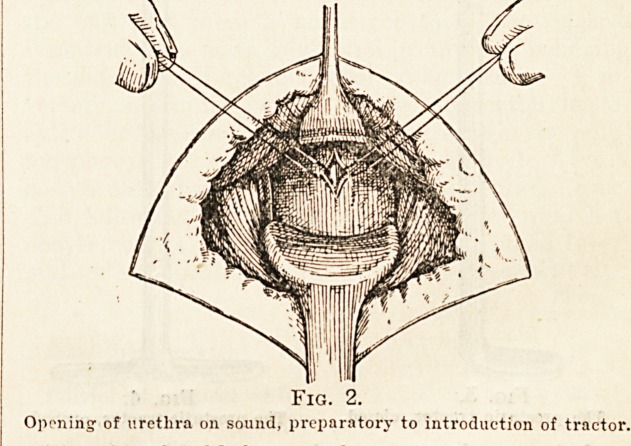


**Fig. 3. f3:**
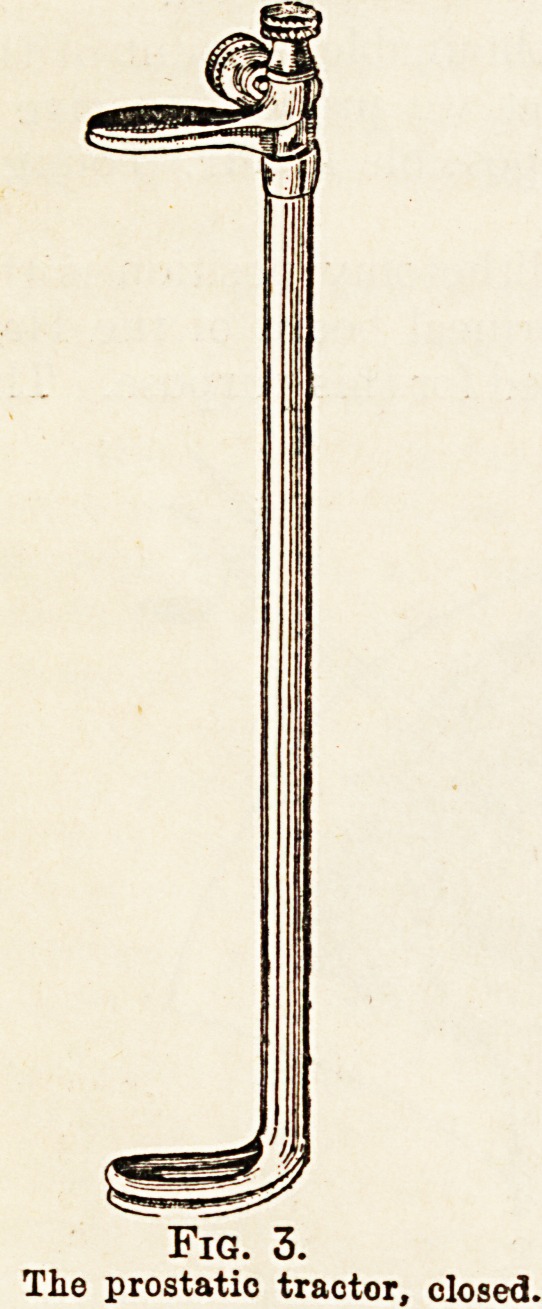


**Fig. 4. f4:**
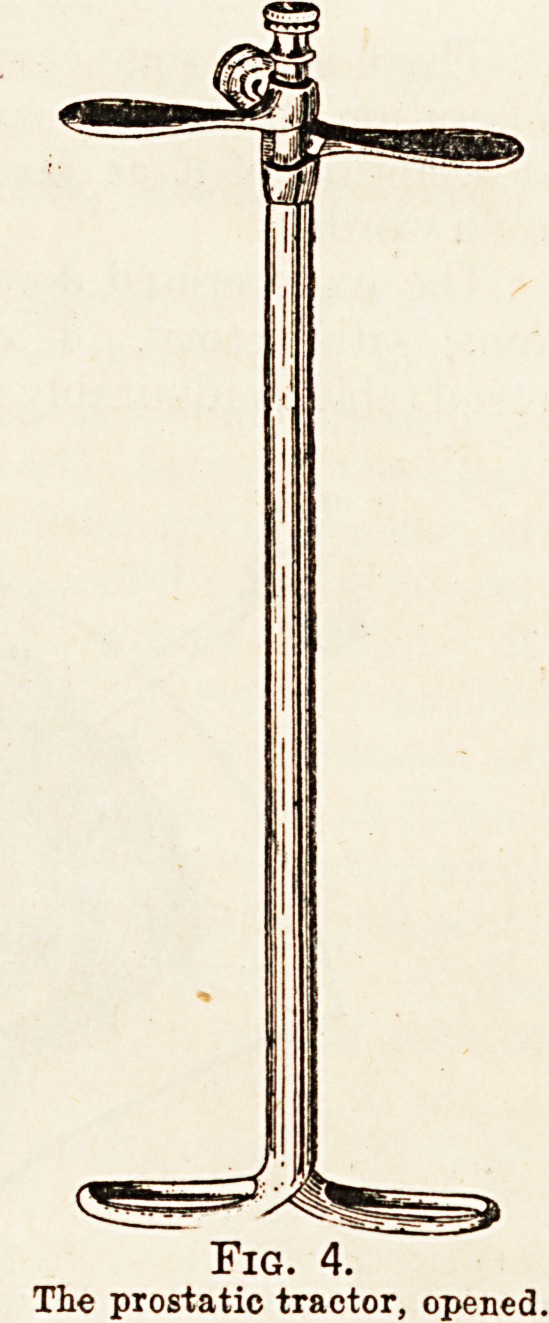


**Fig. 5. f5:**